# Disaster Displacement in the U.S.: Understanding Patterns and Implications for Older Adults

**DOI:** 10.1093/geroni/igaf122.1518

**Published:** 2025-12-31

**Authors:** Jenna Tipaldo, Deborah Balk, Frank Heiland

**Affiliations:** City University of New York, New York, New York, United States; Baruch College, CUNY, New York City, New York, United States; Baruch College, CUNY, New York City, New York, United States

## Abstract

This research project utilizes the new nationally representative, rapid-turnaround Household Pulse Survey (HPS) data to investigate the prevalence and vulnerability of natural disaster displacement in the United States. We analyze data from all HPS cycles from December 2022 through September 2024, focusing on individuals ages 60 and older, making comparisons within groups of older adults as well as to the general population. Key preliminary findings are that 1.2% of the general population reports displacement due to a natural disaster in the past year (asked from late 2022 through Q3 of 2024). Importantly, displacement is not distributed evenly and varies by state – e.g., Florida has a much higher share of disaster displacement and a relatively larger share of older adults – income classes, race/ethnicity, and type of disaster. The oldest adults seem more likely to be displaced due to multiple disasters, and many older adults who are displaced face health hazards and disability: such as food and water shortages, loss of electricity, and unsanitary conditions, isolation and mental health impacts, and both cognitive and physical disabilities. Preliminary results also suggest that working older adults resemble the general population in terms of work disruptions: those who report displacement in the past year are also more likely to report recent loss of income or work. These compositional issues, as well as plausible mechanisms, are also analyzed and discussed.

